# Lichen planopilaris and frontal fibrosing alopecia: review and update of diagnostic and therapeutic features^☆^

**DOI:** 10.1016/j.abd.2021.08.008

**Published:** 2022-04-02

**Authors:** Carolina Oliveira Costa Fechine, Neusa Yuriko Sakai Valente, Ricardo Romiti

**Affiliations:** Department of Dermatology, Faculty of Medicine, Universidade de São Paulo, São Paulo, SP, Brazil

**Keywords:** Alopecia, Diagnosis, Lichen planus, Review, Scalp dermatoses, Therapeutics

## Abstract

Lichen planopilaris and frontal fibrosing alopecia are primary scarring alopecias where diagnosis can be suggested by clinical and trichoscopy features, especially in the early stages, but scalp biopsy is the standard exam for definitive diagnosis. Frontal fibrosing alopecia is considered a variant of lichen planopilaris, as the histopathological findings are similar, with a perifollicular lymphohistiocytic infiltrate, sometimes with a lichenoid pattern. A thorough clinical examination, trichoscopy and photographic documentation are essential to assess the evolution and therapeutic response. To date, there are no validated treatments or guidelines for these diseases, but there are recommendations that vary with the individual characteristics of each patient. This article presents a comprehensive review of the literature, including an update on topics related to the diagnosis, follow-up, histopathological aspects and available treatments for lichen planopilaris and frontal fibrosing alopecia, highlighting their similarities, differences and peculiarities.

## Introduction

**Figure 1:**
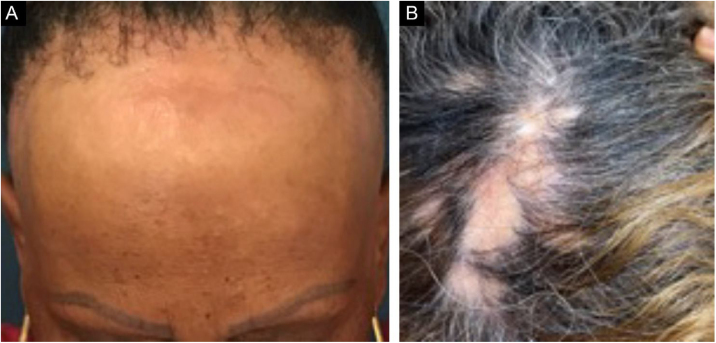
(A), Frontoparietal, eyebrow alopecia and facial papules in the frontal region (FFA). (B), Areas of multifocal alopecia in parietal region and vertex (LPP).

Frontal fibrosing alopecia (FFA) is a primary lymphocytic scarring alopecia, considered a variant of lichen planopilaris (LPP) due to its histopathological. Clinically, FFA causes hair loss that occurs slowly in the frontotemporal implantation hairline, commonly associated with eyebrow alopecia. In addition to the scalp, other manifestations may arise, such as lichen planus pigmentosus, facial papules, body hair involvement, hypochromic lesions, diffuse erythema on the facial and cervical regions, evident frontal veins. On the other hand, classic lichen planopilaris (LPP) shows an area of ​​irregular alopecia that is more common in the vertex region, single or multifocal, and may occur in other places on the scalp, but without a characteristic band distribution (Fig. 1). Graham Little-Piccardi-Lassueur syndrome (GLS) is a rarer subtype of LPP, characterized by scarring alopecia on the scalp, non-scarring hair loss in the axillary and pubic regions, and the appearance of lichenoid follicular papules on the trunk and extremities.

The present article aims to review the diagnostic aspects, inflammatory activity, follow-up, and treatments of the two diseases, highlighting their similarities, differences and peculiarities.

## Diagnosis and follow-up

**Figure 2:**
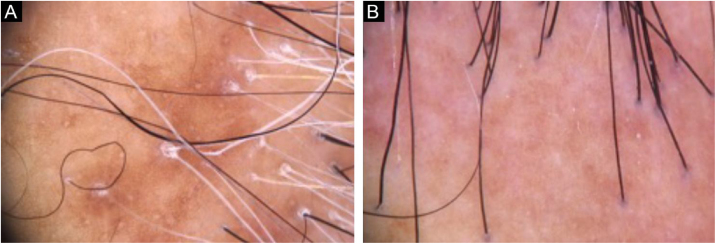
Trichoscopy with findings common to patients with LPP and FFA. (A), Peripilar desquamation and absence of follicular openings in a patient with LPP. (B), Mild peripilar desquamation and mild peripilar erythema in a patient with FFA.

The presence of clinical and trichoscopic findings characteristic of LPP or FFA could replace scalp biopsy in the opinion of some authors.^1^ In scarring alopecia, the individual characteristics of each disease in the initial clinical picture may lead to the diagnostic definition. However, there is an overlap of many clinical findings in different conditions (Fig. 2). The need for a scalp biopsy for dubious cases is unanimous. The site should be chosen with the aid of trichoscopy.^2^ In addition, the histopathological examination is necessary for undeniable documentation of fibrosis and evaluation of the inflammatory process with quantification of the perifollicular infiltrate involved in each disease, which impacts the therapeutic choice, follow-up and prognosis.

A list of diagnostic criteria for FFA was proposed by Vañó-Galván et al. in a 2018 publication.^3^ The major criteria are scarring alopecia of the scalp in the frontotemporal region (in the absence of keratotic follicular papules on the body) and bilateral diffuse alopecia of the eyebrows. The minor criteria include trichoscopy with typical characteristics (peripilar erythema, peripilar desquamation, or both); histopathological characteristics of scarring alopecia with FFA or LPP pattern; involvement of other areas suggestive of FFA (occipital region, face, sideburns, body hair) and presence of non-inflammatory facial papules. The diagnosis requires two major criteria or one major and two minor criteria.

There are no specific laboratory tests for LPP or FFA. Previous studies have demonstrated the occurrence of LPP in chronic inflammatory and autoimmune diseases, as well as a significant prevalence of autoimmune diseases and thyroid abnormalities in FFA patients when compared to the normal population.^1^ A case-control study showed an association of hypothyroidism, rosacea, and LPP in women with FFA, suggesting that tests should be carried out to exclude comorbidities in this group.^4^ There is no consensus on whether to screen all patients with LPP or FFA.^5^

Some disease activity and severity classifications have been proposed for these diseases in recent years.

In 2010, an LPP activity index (LPPAI) was introduced by Chiang et al. for a more objective approach to the clinical course and therapeutic response.^6^ The study included 40 patients with LPP and FFA, 29 with LPP, seven with FFA, and four with both diseases. Up to then, there were no studies with systematic data and evaluation objectives. The index consists of symptoms (pruritus, pain and burning sensation), signs (diffuse erythema, peripilar erythema, peripilar desquamation), pull test (presence of anagen hairs), and progression (patient perception of clinical worsening) - (Table 1). The assessment is performed through a physical examination without trichoscopy. The pull test is considered positive for scarring alopecia when some anagen hairs are collected. The authors assigned numerical values ​​to these subjective and objective markers to obtain a quantitative summary of disease activity, the LPPAI.^6^

**Table 1:** Lichen Planopilaris Activity Index (LPPAI).^6^

**LPPAI (0-10)** = (pruritus + pain + burning sensation)/3 + (diffuse erythema + perifollicular erythema + perifollicular desquamation)/3 + 2.5 (pull test) + 1,5(progression)/2
Signs and symptoms: 0 = absent, 1 = mild, 2 = moderate, 3 = severe.
Pull test 0 = no anagen and 1 = any anagen.
Progression 0 = equal, 1 = indeterminate, 2 = worsening.

Adapted from: Chiang C, et al. 2010.^6^

In 2016, Holmes et al. proposed the FFA severity index (FFASI) as an assessment method for clinical practice and scientific publications (Table 2). The FFASI is compiled in two ways: A and B. The FFASI-A uses clinical images of the implantation hairline divided into four sections. The alopecia severity is graded from 1 to 5, based on the advancement of the implantation hairline. In addition to the extent of the affected area, loss of eyebrows, extra-facial alterations such as loss of body hair, changes in mucous membranes, presence of lichen planus pigmentosus, among other findings, which are scored as: no loss, partial loss and total loss, and present or absent, respectively. All the scores can be combined to obtain a maximum score of 100. The FFASI-B assesses the same characteristics, but includes scores to assess inflammation and density, as well as the involvement of other body areas.^7^ The study suggested the registration in a form for global disease follow-up and therapeutic response comparison. The most relevant criterion, considered according to the authors experience, was the extension and clinical evolution of the area of alopecia.

**Table 2:** Frontal Fibrosing Alopecia Severity Index (FFASI).

**Extension**	Grade 1: = or <1 cm;
	Grade 2: 1 to 2.9 cm;
	Grade 3: 3 to 4.9 cm;
	Grade 4: 5 to 7.9 cm;
	Grade 5: = or >8cm
**Scalp margin**	Grade 1 to 5
	(No loss = score 0; Grade 1 = score 4;
	Grade 2 = score 8; Grade 3 = score 12;
	Grade 4 = score 16; Grade 5 = score 20)
Frontal	
Right Lateral	
Left Lateral	
Posterior	
**Frontal Band**	score 0 = no inflammation and normal density;
	score 2 = inflammation or density reduction;
	score 4 = inflammation and density reduction;
Total	/84
**Alopecia in other types of hairs**	Absent = score 0
	Partial loss = score 1
	Total loss = score 2
Eyebrows	
Lashes	
Flexural (armpits, pubic)	
Upper limbs	
Lower limbs	
**Other findings**	Absent = score 0
	Present = score 1
Classic LPP	
Facial papules	
Cutaneous LP and variants	
Oral mucosa LP	
Genital mucosa LP	
Ungual LP	
Total	/16
Combined total	/100

LPP, Lichen planopilaris; LP, Lichen planus.

Adapted from: Holmes S, et al. 2016.^7^

Some authors criticize the FFASI scoring system for considering it complex for clinical practice, with criteria that do not properly represent the FFA prognosis and that lack validation. As a result, in 2018, a new FFA severity score (FFASS) was proposed that included clinical features, such as the extent of frontotemporal alopecia, loss of eyebrows, peripilar erythema, peripilar desquamation, pruritus, and pain (Table 3). The severity score ranged from 0 to 25 (with the highest value being the most severe).^8^ Although FFASS is simpler for practical application when compared to FFASI, it also has limitations. There are no trichoscopy data and all patients evaluated were females.

**Table 3:** Frontal Fibrosing Alopecia Severity Score (FASS).^8^

**Clinical signs**	
1. Scalp alopecia (band-shaped scar measurement)	
Regions:	__/10
Frontal	__/5
Left temporal	__/5
Right temporal	
<1 cm (1) 1-2.99 cm (2) 3-4.99 cm (3) 5-6.99 cm (4) >7 cm (5)	
2. Loss of eyebrows	
No (0) Partial (0,5) Total (1)	__/1
Extension	__/21
3. Peripilar inflammation	
A. Intensity	
Peripilar erythema: Absent (0), mild (0,1), intense (0,2)	__/0.2
Peripilar desquamation: Absent (0), mild (0,5), intense (1)	__/1
B. Extension to the frontotemporal line	
Peripilar erythema: <25% (0); 25%-75% (0.1); >75% (0.2)	
Peripilar desquamation: <25% (0); 25%-75% (0.5); >75% (1)	
**Associated symptoms**	
1. Pruritus	__/0.2
Intensity and frequency: absent (0); mild or occasional (0.1), intense	
2. Pain	__/0.6
Intensity and frequency: absent (0); mild or occasional (0.3), intense or daily (0.6)	
Degree of inflammation	__/4
Total FFASS score	__/25

Adapted from: Saceda-Corralo D, et al. 2018.^8^

In our clinical practice, it has been observed that many patients being followed for LPP and FFA, without inflammatory symptoms or signs, continue with progression of the area of alopecia. On the other hand, a study with 62 women with FFA showed that one in four patients undergoing treatment, even with disease stability, had persistent symptoms and inflammatory signs. Moreover, some patients had worsening and progression of alopecia without inflammatory signs, suggesting that inflammatory infiltrates restricted to the isthmus are not visible on the skin surface.^9^

**Figure 3:**
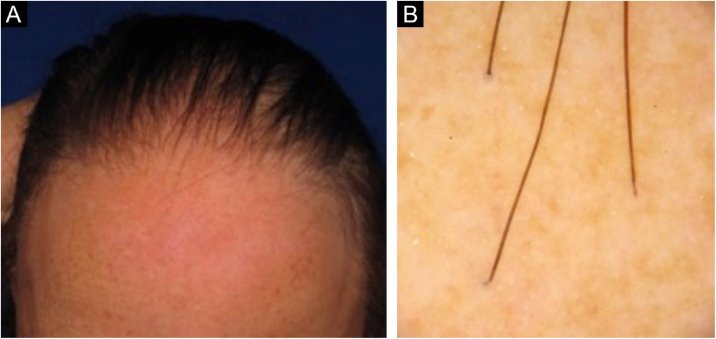
(A), Frontotemporal alopecia in a patient with FFA and absence of eyebrows. (B), Trichoscopy: mild peripilar desquamation and absence of follicular openings.

**Figure 4:**
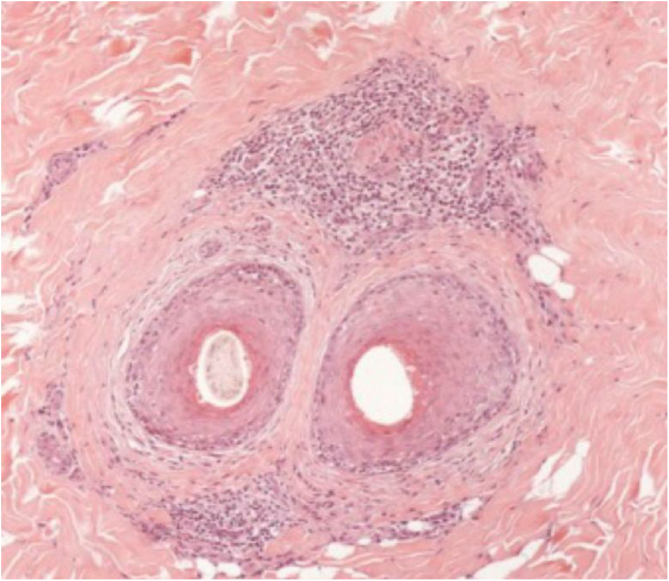
Histopathological: cross-sectionning (Hematoxylin & eosin, ×100). Presence of moderate perifollicular lymphocytic inflammatory infiltrates in the isthmus region.

Figure 3, Figure 4 show the clinical images of the frontotemporal region, trichoscopy, and histopathology of a patient with FFA from the specialized care center where our study was carried out, with no symptoms or signs on physical examination, with mild peripilar desquamation on trichoscopy, but with significant lichenoid inflammatory infiltrate in the isthmus and the lower hair portion on histopathology.

The degree of FFA progression is difficult to predict, but some studies mention factors related to prognosis. A clinical pattern of diffuse involvement of the frontotemporal recess, loss of eyelashes, loss of body hair, and presence of facial papules were indicators of a poor prognosis.^1^, ^10^ On the other hand, a clinical pattern of pseudo fringe, mild involvement of eyebrows, and younger age of onset were all associated with a less severe course of the disease.^10^, ^11^

## Histopathological features

A scalp biopsy allows the diagnostic confirmation of scarring alopecia. It must be performed with a 4-mm punch biopsy, with one sample submitted for vertical sections and the other for cross-sectional sections. The cross-sectional or horizontal sections ensure the observation of all follicles in the sample at different depth levels. The vertical or longitudinal sections are appropriate for the evaluation of alopecias with pathological alterations in the epidermis and dermo-epidermal interface (e.g., lupus erythematosus).^12^

**Figure 5:**
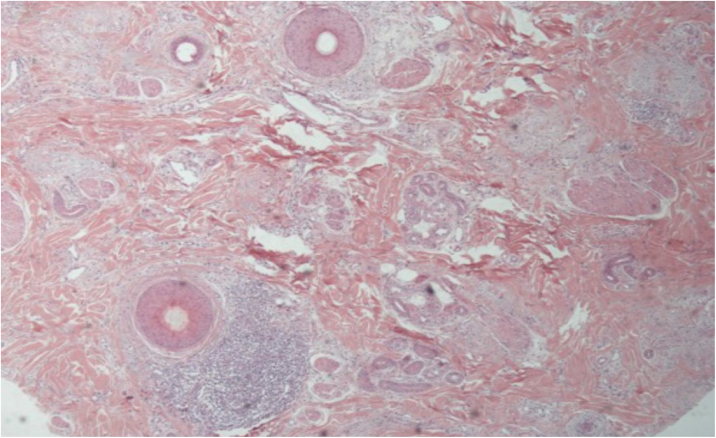
Histopathological: cross-sectionning (Hematoxylin & eosin, ×100) – Presence of perifollicular lymphocytic inflammatory infiltrate in the isthmus region and concentric eosinophilic fibrosis.

The histopathological alterations found in LPP are: a perifollicular lymphohistiocytic infiltrate, sometimes with a lichenoid pattern, more prominent in the upper portion (isthmus and infundibulum regions); vacuolar degeneration of basal cells, necrotic keratinocytes, and artifactual clefts between the follicle and the perifollicular fibrous band; perifollicular fibrosis can be seen separating the inflammatory infiltrate from the follicle (Fig. 5). Over time, there is a reduction and loss of sebaceous glands and destruction of the entire hair follicle, which is replaced by connective tissue.^13^

Direct immunofluorescence (DIF) is used to detect antibody deposits in lesional and perilesional tissue. It has shown to be useful in the differential diagnosis of some scarring alopecias such as LPP and lupus erythematosus. When DIF is positive in LPP, it shows a pattern of globular deposits, cytoid bodies (generally IgM) clustered adjacent to the follicular epithelium, irregular fibrinogen deposition, and C3 deposits in the papillary dermis. A linear band of immunoreactivity (IgG, IgM, and C3) is typical of lupus erythematosus (LE).^13^

FFA is considered a variant of LPP because it shows indistinguishable histopathological findings (Fig. 5). In the scientific literature, a few studies have compared the characteristics of each disease.

Poblet et al. (2006) described eight cases of each disease, also using DIF in two cases of FFA and in four cases of LPP. They found more prominent inflammatory infiltrate and fewer apoptotic cells in LPP than in FFA, but it was not enough to consider these diseases distinct concerning their histopathology. Regarding DIF, the result was negative for FFA and positive for two cases of LPP.^14^

Cerqueira et al. (2016) compared ten cases of LPP and FFA, also using DIF and immunohistochemical markers (CD1a, CD3, CD4, CD8, CD68 and indolamine-2,3-dioxygenase). No significant differences were observed between the diseases.^15^

Donati et al. (2017) evaluated the DIF results in patients with FFA and found 29.5% of positive results, with 9% showing a pattern similar to LPP, 7% an LE pattern, and 13.5% non-specific patterns. The study concluded that the DIF of FFA does not have the same diagnostic value as in LPP and LE, being, in most cases, negative and without a specific pattern.^16^

Wong and Goldberg (2017) compared 32 cases of LPP and 37 cases of FFA. They found statistically significant differences between the extent of the inflammatory infiltrate below the isthmus in FFA when compared with LPP (92% *versus* 63%; p = 0.02) but found no differences between the inflammatory infiltrate intensity in the two diseases.^17^

Gálvez-Canseco et al. (2018) analyzed 20 histopathological parameters in 34 patients with LPP and 44 with FFA; however, they only observed three statistically significant histopathological parameters. The presence of terminal catagen telogen hairs (23.5% LPP *versus* 50% FFA; p = 0.020); intense perifollicular peripheral inflammatory infiltrate (29.4% LPP *versus* 4.6% FFA; p = 0.010) and concentric lamellar fibroplasia zone (85.3% LPP *versus* 63.6% FFA; p = 0.041).^18^ The authors did not perform immunofluorescence or immunohistochemistry, as they stated that most histopathological diagnoses of alopecia are based on routine hematoxylin & eosin staining.

Pindado-Ortega et al. (2020) performed a study to analyze histopathological, immunohistochemical (CD4, CD8, FOXP3), and hormone receptor profile (ER-b estrogen beta and androgen receptor) findings in affected and unaffected areas of the scalp of patients with FFA. Perifollicular inflammatory infiltrate was observed in both areas, but with greater intensity in the affected site. FOXP3+ regulatory T (Treg) cells were paradoxically increased in the affected areas when compared to the healthy scalp. There were no differences in relation to the hormone receptor profile.^19^

However, a subsequent study with immunohistochemical analysis indicated a significant difference between LPP and FFA, with a higher number of CD68+ cells in the LPP samples. There was also a decrease in CD86 expression and an increase in CD163 and IL-4 expression in lesional LPP when compared to FFA samples. The data suggested that macrophages exist in different functional phenotypes in the two diseases (increased M2 phenotype in lesional LPP), with emphasis on CD86 and CD163 as potential markers to differentiate between the conditions.^20^ The study corroborated the increase in cytotoxic CD8 cells in the bulge and infundibulum region of patients with LPP and FFA, but with no differences between the diseases.

To date, although some histopathological features have been shown to be different between FFA and LPP, the differences are considered subtle or non-specific to reliably differentiate them. Therefore, as in previous studies, clinical correlation is essential for the differential diagnosis between the diseases.

## Treatment

The goal of treating LPP and FFA is to prevent progression of the scarring alopecia area​​ and to improve symptoms. The therapeutic choice will depend on several factors such as: age of the patient, location of the clinical lesion, stage of the disease, presence of symptoms, comorbidities, and histopathological inflammation.

The management of these diseases is challenging. The level of evidence for current therapies is weak, with case reports, case series, expert opinions, and a few controlled studies. There are some recommendations, but to date, there are no specific treatment guidelines for LPP and FFA.

### Therapeutic approach in LPP

Intralesional and topical corticosteroids are considered the first-line treatments for LPP.^21^ They can be used as monotherapy or associated with other drugs with varying degrees of success. They are indicated in localized lesions of a limited extent. Triamcinolone acetonide at a concentration of 10 mg/mL, totaling 2 mL, every 4 to 6 weeks is recommended for the treatment of LPP at the University of British Columbia.^22^ The observed effect is the improvement of inflammatory signs and symptoms, with attention being paid to the risk of scalp atrophy. The recommendation is to maintain the treatment until the disease stabilizes. If there is no improvement after three months, other treatment options should be evaluated.

High potency topical corticosteroids can be used together with intralesional therapy for a faster clinical response. Clobetasol propionate lotion or cream, up to twice a day, is a therapeutic alternative. Lyakhovitsky et al. reported a low success rate in patients treated with topical corticosteroids (eight responders of 42 patients on monotherapy), whereas they observed better results with intralesional corticosteroids (11 responders of 15 patients on monotherapy).^23^ In turn, Mehregan et al. had a higher success rate in patients treated with topical and oral steroids than those treated with intralesional steroids.^24^

Oral corticosteroids are reserved for more extensive manifestations, very symptomatic patients and those with rapid progression. Prednisone at a dose of 1 mg/kg/day can be administered with dose adjustment after one month. There is also the alternative of temporary use, due to the rapid mechanism of action, acting as a bridge to the effect of longer-lasting drugs such as methotrexate.^25^ Despite the good therapeutic response, the recurrence rate is high and adverse effects can be important.

Hydroxychloroquine is used in the treatment of LPP, especially in more extensive cases. Although its exact mechanism of action on the disease is uncertain, it is believed to be related to antigen presentation, cytokine production, and stimulation of toll-like receptors 9.^26^ Action onset occurs after two to three weeks, peaking after six months. A review by the American Academy of Ophthalmology recommends a maximum daily dose of 5 mg/kg.^27^ A previous ophthalmologic evaluation is required, with an eye fundus examination, biochemical evaluation with complete blood count, liver function, screening for glucose 6-phosphate dehydrogenase (G6PD) deficiency, especially in male patients. Adverse reactions are rare but may include skin hyperpigmentation, retinopathy, abdominal pain, anorexia, nausea, myalgia, and hematological alterations. Caution is required in women of childbearing potential, as the risks to the fetus include neurological disorders and interference with hearing, balance, and vision.

There is a randomized clinical trial evaluating the use of hydroxychloroquine (400 mg daily) *versus* methotrexate (15 mg weekly) administered for six months in refractory cases of LPP.^28^ There was a significant decrease in LPPAI in the second and fourth months of hydroxychloroquine use, but methotrexate was more effective in reducing the average LPPAI in the total period.

Other studies have reported success rates with the use of hydroxychloroquine, ranging from 40.1% to 76%.^6^, ^23^, ^29^ However, there are data from small series or case reports with disappointing results reported for the same drug.^24^

Immunomodulators are therapeutic alternatives in patients with the difficult-to-control disease. Some options pointed out in the literature are cyclosporine, 3-5 mg/kg/day; mycophenolate mofetil, 500 mg twice daily; and oral methotrexate, 15 mg/week.^25^, ^28^

Several different treatment modalities are reported for refractory LPP, with inconsistent results. Some of them are systemic retinoids, tetracyclines, thalidomide, dapsone, pioglitazone, topical calcineurin inhibitors, griseofulvin.^21^, ^25^, ^30^

Isotretinoin and oral acitretin have shown beneficial effects on generalized and erosive lichen planus, which led some authors to expand their use to LPP. However, discreet results and high recurrence rates were observed after discontinuation. Common side effects are xerosis, cheilitis, conjunctivitis.^22^, ^25^

Studies on the use of tetracyclines for LPP are disappointing. Spencer et al. reported that only 27% of patients treated with oral doxycycline (200 mg/day for three to six months as monotherapy) had positive responses.^29^ Similar results were found by other authors.^23^, ^24^

Topical calcineurin inhibitors have demonstrated a beneficial effect in inducing the anagen phase in animal models.^31^ However, the largest study of topical calcineurin inhibitors, in ten patients with LPP, reported inflammation improvement in only two patients (one on monotherapy and one associated with hydroxychloroquine).^23^

In recent years, Janus kinase (JAK) inhibitors have been studied especially in the treatment of alopecia areata, as they are small molecules involved in inflammatory signaling pathways, reducing inflammation. The effect of tofacitinib, a pan-Janus kinase inhibitor (preferentially for JAK1/JAK3), was retrospectively evaluated by Yang et al. in 10 patients with recalcitrant LPP (8 classic LPP and 2 FFA). The dose used was 5 mg orally, two to three times a day for two to nineteen months as monotherapy or adjuvant therapy. Eight of ten patients showed improvement (4 as monotherapy and 4 as adjunctive therapy). The reduction in the LPPAI activity index ranged from 30 to 94%. As an adverse effect, only one patient complained of weight gain.^32^

Another recently studied drug was oral minoxidil. A retrospective study evaluated the impact of its use in 51 patients with classic LPP, with a dose ranging from 0.25 to 1 mg/day. There was an increase in hair thickness in the unaffected areas adjacent to the alopecia, leading to better coverage.^33^

The challenge in the treatment of LPP was evidenced in a retrospective study, demonstrating that 42.9% of a total of 261 patients with classic LPP required drugs from three different classes, and 11.3% did not achieve a complete response. All patients received topical corticosteroids as monotherapy or combined with systemic agents. Among the relatively effective drugs with total or partial response were: cyclosporine 100% (in a total of 16 patients); methotrexate 85% (in 26 patients); mycophenolate mofetil 76% (in 66 patients); prednisolone 68% (in 22 patients); intralesional corticosteroid 43% (in 7 patients); and hydroxychloroquine 59% (in 68 patients). The median time to achieve complete and partial remission was 6.1 ± 6.5 and 2.7 ± 3.9 months, respectively.^34^

### Therapeutic approach in FFA

FFA is a chronic disease with a variable course and possible spontaneous stabilization, which often makes it difficult to assess the effectiveness of treatment modalities.

Topical therapy recommendations in the literature include high-potency corticosteroids, intralesional corticosteroids, and calcineurin inhibitors. The main systemic treatments are 5 alpha-reductase inhibitors, hydroxychloroquine, and retinoids. Some studies report the use of tetracyclines, methotrexate, pioglitazone, naltrexone, and JAK inhibitors (tofacitinib).

Intralesional corticosteroid use (triamcinolone acetonide) is one of the most commonly used therapeutic options in FFA, especially for lesions in well-delimited areas, implantation hairline, and eyebrows.^35^ In a retrospective study, Banka et al. demonstrated disease stabilization with monthly injections of triamcinolone acetonide 2.5 mg/mL in the frontal region after 4 to 5 sessions.^36^ The first published study with eyebrow infiltration was performed at a concentration of 10 mg/mL with 0.125 mL per eyebrow, showing positive results.^37^ However, the current recommendation is the use of lower concentrations (2.5 mg/mL), both on the scalp and on the eyebrows, due to the risk of skin atrophy and inaesthetic results.^5^ The therapeutic approach to eyebrow alopecia in FFA follows the treatment of the disease and should be considered between the topical and systemic options according to the involvement of the disease, whether symptomatic, localized, or extensive.

Topical high-potency corticosteroids and topical calcineurin inhibitors have shown beneficial effects in improving symptoms but little effect in controlling disease progression.^5^, ^35^, ^38^ A retrospective study showed improvement of pruritus in 44%, trichodynia in 33%, and stabilization in 64.6% of patients with the combined use of a high-potency topical corticosteroid and a topical calcineurin inhibitor.^39^ In practice, these agents are combined with other systemic therapies. Some authors criticize the use of topical corticosteroids, claiming a risk of worsening skin atrophy, a characteristic of FFA, and an increase in telangiectasias.^40^

Topical minoxidil 5% can be used to improve hair coverage on the scalp, especially in patients with associated androgenetic alopecia. Additionally, an *in vitro* study demonstrated that minoxidil significantly reduced the total amount of collagen (30% inhibition) in cultured rat dermal papilla cells, with a possible antifibrotic effect.^41^ Controlled studies in humans are necessary to evaluate this effect in scarring alopecias.

FFA is currently understood as a generalized disease that affects all body hair and is associated with several skin alterations, such as papules on the face and lichen planus pigmentosus. Therefore, systemic therapies are often necessary.

The 5-alpha-reductase inhibitors (finasteride and dutasteride) are systemic drugs that have shown positive results in the treatment of FFA, even in patients without associated androgenetic alopecia.^42^ Some authors state it is the most beneficial therapeutic modality for FFA.^35^ It is believed that these substances act on the etiology of the disease, with a probable hormonal mechanism.

There is a case report showing improvement in inflammatory signs, cutaneous atrophy, and repilation in the frontotemporal region of a patient with FFA after the introduction of finasteride 2.5 mg/day.^43^ In the first multicenter study of FFA with 355 patients, the use of finasteride 2.5 to 5 mg/day or dutasteride 0.5 mg/week showed disease stabilization or improvement in all patients; however, the study design was retrospective, and many patients were on combined therapies.^1^

A review study on the efficacy and safety of 5 alpha-reductase inhibitors for FFA reported only two publications with a moderate level of evidence; lack of dose standardization (finasteride 2.5 to 5 mg/day, dutasteride 0.5 mg 1 to 3 times a week or daily use with a mean time of use of one year); combined use with other therapeutic agents; positive results in recalcitrant disease. The use of 5 alpha-reductase inhibitors was considered positive due to the good results, with a reduction in disease progression. There is limited evidence on the safety profile. The use in women of childbearing age should be cautious due to teratogenicity. Although some authors have reported a protective effect in breast cancer,^44^ it is best to avoid its use in women with a personal or family history of breast cancer until more data are available.

A recent retrospective observational study on the use of oral dutasteride in FFA in 224 patients with a mean follow-up of two years showed that the medication was the most effective therapy compared to other systemic therapies or no systemic treatment. The stabilization rate for the frontal, right, and left temporal regions after 12 months was 62%, 64%, and 62% in the dutasteride group (n = 148), 60%, 35%, and 35% with other systemic therapies (n = 20) and 30%, 41% and 38% without systemic treatment (n = 56; p = 0.000, 0.006 and 0.006, respectively). The stabilization showed a statistically significant association with an increasing dose of oral dutasteride with a weekly treatment of 5 to 7 doses of 0.5 mg. The medication was well tolerated in all patients.^45^ But there are limitations in the study, such as the retrospective design and a small number of patients treated with other drugs.

The main targeted therapies against the lymphocytic inflammation of FFA are hydroxychloroquine and doxycycline. These drugs have anti-inflammatory properties and an acceptable adverse effect profile.^35^ Chiang et al. evaluated the efficacy of hydroxychloroquine 400 mg/day on LPP and FFA using the LPPAI activity index. There was a positive effect on both diseases, with an improvement of signs and symptoms in 69% of patients after six months (71% in patients with FFA) and 83% after one year (50% in patients with FFA).

Studies using doxycycline 100 to 200 mg/day have shown inconsistent results. A retrospective study reported 50% improvement in signs and symptoms of FFA *versus* 50% with no response after one year.^46^ The most common adverse effect is gastrointestinal alterations (nausea, vomiting, and gastroesophageal reflux).

Systemic retinoids are also thought to have an anti-inflammatory effect on FFA and may lead to normalization of the antigenic expression of follicular keratinocytes, but the exact mechanism of action remains unknown. The retrospective cohort study by Rakowska et al. evaluated groups treated with oral isotretinoin 20 mg/day and oral acitretin 20 mg/day and compared them with the group treated with oral finasteride 5 mg/day. Disease stabilization was observed in 79% in the isotretinoin group, 73% in the acitretin group *versus* 43% in the finasteride group. And the response was maintained one year after drug discontinuation (72% isotretinoin group, 73% acitretin group, and 43% finasteride control group).^47^

Retinoids have also shown beneficial effects in the treatment of facial papules and lichen planus pigmentosus in studies using oral isotretinoin 20 mg/day.^48^, ^49^ It is important to carefully evaluate administration in women of childbearing age and to perform biochemical evaluations before and during treatment.

PPAR-gamma agonists (pioglitazone) have been used in FFA, but with variable results and many adverse effects. A retrospective cohort study reported disease stabilization in three of four patients, but the evaluation was carried out for a short period, one to three months, and in a small sample. The main adverse effects were edema of the extremities, weight gain, and dizziness.^50^ Other medication-associated effects are heart failure, increased risk of bladder, prostate, and pancreas cancer.^5^

Naltrexone is an opioid antagonist with anti-inflammatory properties and an alternative treatment, at low doses, for scarring alopecia. It seems to be able to reduce symptoms, inflammatory signs, and disease progression.^51^ It is important to investigate the presence of mood disorders, liver function, and the use of other medications before starting the treatment. The reported adverse effects are abdominal pain, nausea, headache, and anxiety.

Immunosuppressive agents can be used in refractory cases of FFA, but there are scarce data in the literature and inconsistent results.

Gerkowicz et al. evaluated the effectiveness of diode laser (LED) devices as an adjuvant treatment in scarring alopecia.^52^ The LPPAI activity index and the FFASS severity index were significantly reduced after treatment (p = 0.012, p = 0.017, respectively). Within the treated area, there was an improvement in hair shaft thickness with an increase in the number of terminal hairs (p = 0.009), while the number of intermediate and thin hairs did not change significantly (p = 0.836, p = 0.675, respectively). The treatment with LED was found to be safe and well-tolerated, with symptoms reduction in both FFA and LPP.^52^

Hair transplant surgery has been considered an optional approach for cases with a high impact on the patients quality of life. Some points that should be considered are: clinical stabilization does not necessarily imply disease remission; reactivation can lead to graft loss; the surgical procedure may be responsible for the reactivation of the disease (Koebner phenomenon).

A retrospective multicenter study evaluated 51 patients with FFA submitted to hair transplant surgery, performed after a mean of 15 months of stabilization (according to clinical criteria and trichoscopy). The follicular unit extraction technique was performed in 14% of the cases and the band technique in 86% of the cases. The most frequent locations were: temporal region (59%), frontal region (44%), and eyebrows (29%). All patients maintained the drug treatment for FFA after transplantation. The mean graft survival after 1, 2, 3, and 5 years of follow-up was 87%, 71%, 60%, and 41%, with a significant decrease after five years. However, the patient satisfaction rate was 82%.^53^

## Conclusion

FFA is considered a variant of LPP due to the histopathological similarities, but with several peculiarities ranging from clinical aspects to variations in therapeutic response. The scientific literature suggests that, to date, there are no histological or immunological findings that allow us to accurately differentiate these two forms of primary scarring alopecia.

The best method for evaluating the inflammatory process, which allows proving undeniable evidence of fibrosis, is the histopathological examination. Moreover, inflammation at the isthmus level is not fully translated by the clinical signs and symptoms described for these diseases, which can impact the therapeutic decision.

The absence of randomized clinical trials does not allow drawing definitive conclusions about the most effective of the available treatments. Furthermore, most studies evaluate patients with combined treatments, so the best choice will be made on a case-by-case basis. The therapeutic approach to LPP and FFA may also vary with patient evolution and follow-up. Therefore, photographic documentation and trichoscopy are essential to assess disease progression or stabilization.

## Financial support

None declared.

## Authors’ contributions

Carolina Oliveira Costa Fechine: Approval of the final version of the manuscript; design and planning of the study; drafting and editing of the manuscript; collection, analysis, and interpretation of data; intellectual participation in the propaedeutic and/or therapeutic conduct of the studied cases; critical review of the literature; critical review of the manuscript.

Neusa Yurico Sakai Valente: Approval of the final version of the manuscript; effective participation in research orientation; intellectual participation in the propaedeutic and/or therapeutic conduct of the studied cases; critical review of the manuscript.

Ricardo Romiti: Approval of the final version of the manuscript; effective participation in research orientation; intellectual participation in the propaedeutic and/or therapeutic conduct of the studied cases; critical review of the manuscript.

## Conflicts of interest

None declared.
